# Autonomous Visual Navigation of an Indoor Environment Using a Parsimonious, Insect Inspired Familiarity Algorithm

**DOI:** 10.1371/journal.pone.0153706

**Published:** 2016-04-27

**Authors:** Douglas D. Gaffin, Brad P. Brayfield

**Affiliations:** Department of Biology, University of Oklahoma, Norman, Oklahoma, United States of America; Lund University, SWEDEN

## Abstract

The navigation of bees and ants from hive to food and back has captivated people for more than a century. Recently, the *Navigation by Scene Familiarity Hypothesis* (NSFH) has been proposed as a parsimonious approach that is congruent with the limited neural elements of these insects’ brains. In the NSFH approach, an agent completes an initial training excursion, storing images along the way. To retrace the path, the agent scans the area and compares the current scenes to those previously experienced. By turning and moving to minimize the pixel-by-pixel differences between encountered and stored scenes, the agent is guided along the path without having memorized the sequence. An important premise of the NSFH is that the visual information of the environment is adequate to guide navigation without aliasing. Here we demonstrate that an image landscape of an indoor setting possesses ample navigational information. We produced a visual landscape of our laboratory and part of the adjoining corridor consisting of 2816 panoramic snapshots arranged in a grid at 12.7-cm centers. We show that pixel-by-pixel comparisons of these images yield robust translational and rotational visual information. We also produced a simple algorithm that tracks previously experienced routes within our lab based on an insect-inspired scene familiarity approach and demonstrate that adequate visual information exists for an agent to retrace complex training routes, including those where the path’s end is not visible from its origin. We used this landscape to systematically test the interplay of sensor morphology, angles of inspection, and similarity threshold with the recapitulation performance of the agent. Finally, we compared the relative information content and chance of aliasing within our visually rich laboratory landscape to scenes acquired from indoor corridors with more repetitive scenery.

## Introduction

Navigation is critically important to animals. Food and water acquisition, mating, and predator and environmental stress avoidance are essential tasks that select for navigational adaptations. In particular, the mechanisms underlying insect navigation have drawn extensive interest, given the small capacity of the insect brain. For example, honeybees use multiple strategies including learning flights, path integration, guidance by polarized light, and landscape cues to navigate between foraging and nest sites with high precision [1–7].

Beyond providing insights that apply broadly to other homing animals, understanding these navigational strategies could help improve navigation of unmanned autonomous vehicles (UAVs), enhance disaster relief efforts, and augment human sensory defects. Historically, the use of the satellite global positioning system (GPS) has been useful in guiding UAVs to and from specific destinations. However, this technology has significant limitations. For example, navigating inside buildings, through forests or caves, under water, or through disrupted areas is difficult or impossible using GPS information. Furthermore, significant resources and infrastructure are needed to maintain and access GPS information. Many coveted UAV characteristics, such as faithful recapitulation of specific navigational routes, processing of novel stimuli, obstacle avoidance, enhanced performance with experience, and lightweight, energy-efficient CPUs already exist in navigating insects such as bees and ants [8]. While no clear consensus exists on specific mechanisms or strategies used by navigating insects, recent work suggests that mechanisms modeled after their behavior and physiology could help guide UAVs [9–11]. Many sophisticated strategies for goal directed robotic navigation have been studied, such as use of stereo and/or panoramic visual sensors, optic flow, use of reference points, and sensor fusion (reviewed in [12]). Another such strategy that has been implemented extensively is simultaneous localization and mapping, or SLAM (reviewed in [12, 13]). This strategy uses several technologies to create a map of an area, topologically and/or geometrically, while determining place recognition within that map to guide navigation.

A more recent idea on insect navigation that does not require landmark learning, positional awareness, or sequence memorization, is the *Navigation by Scene Familiarity hypothesis* (NSFH) [14]. It draws inspiration from several aspects of insect biology, such as their dense arrays of ommatidia, relatively low-resolution visual sensors, panoramic field of view, and saccadic movement during travel. NSFH suggests that the inherent information content of natural scenes can guide movements along previously traversed routes. The NSFH was built on work begun by Zeil et al., which demonstrated that referenced panoramic images of natural scenes could serve as visual attractors when compared pixel-by-pixel to other nearby images [15]. Navigation using this model is based on the idea that image difference values decrease smoothly as you get nearer to the reference images. The resulting gradient of image differences generates a “catchment area” that can be used to navigate to a reference point or path.

Work by Zeil et al. was conducted over short distances (less than 1 meter), but ants and bees can navigate hundreds of meters or even several kilometers from the nest. Inspired by the panoramic views typical of ants [16], a simple algorithm based on NSFH was used to precisely guide robots and simulated agents over longer distances [14, 17]. The objective was to test whether a simple view classification strategy could guide an agent by moving in the direction that was considered most “familiar”. In one study, a gantry robot collected a series of 360-degree panoramic images in an indoor arena with varying amounts of clutter [17]. By using a simple feature classifier on images learned and those currently seen, and maximizing the confidence value between them during an image scanning protocol, the robot could move in the direction that kept it on the learned path. A subsequent study used this approach to recapitulate learned routes within a simulated environment based on an ant’s visual world [14]. In this egocentric strategy there is no need to learn where one is within a route; the only requirement is to detect which direction seems most familiar as compared to the trained path. The environmental information accesses the appropriate stored neural engram and links the appropriate scene sequence. We recently used a scene familiarity based approach to develop a simple algorithm for recapitulating complex routes in a visual information landscape obtained from 2D images from a downward facing field of view (satellite) set to approximately 250 meters above the Earth [18]. This approach successfully recapitulated routes using visual information not only from complex landscapes such as cities, but also from areas with much less visual information such as lakes and deserts.

In previous work, successful recapitulation of learned routes in simulated scenes was related to several factors, including sensor resolution, distance of objects/information from sensor, degree of navigational rotation, and the amount of visual information within the landscape [15, 16, 18–20]. However, there is a dearth of information detailing the interplay between these factors and navigation success in natural environments. An analysis of this interplay could improve the performance and path recapitulation of UAVs and other robots in a variety of environments. One such analysis used the SeqSLAM algorithm and sought to find the minimal amount and quality of visual information required for place recognition [21]. Rather than scene familiarity, the authors used an image difference function focused on image sequences, a topological map, and matching route segments. Recently, a scene familiarity based algorithm was used in a simulated environment to analyze the influence of sensor resolution and field of view on accurate heading calculation during navigation [22].

An important premise of the NSFH is that the environment from an insect’s or UAV’s perspective contains adequate visual information to avoid aliasing between different views. Numerous studies have shown that cluttered outdoor and indoor environments contain sufficient visual information to facilitate vision-based navigation (reviewed in [12]). These studies have primarily used algorithms that relied not on scene familiarity, but rather on landmarks, reference images, or map generation for route recapitulation. Recently we have shown that 2D representations of outdoor scenes (acquired from satellite images) contain robust visual information for a simulated aerial sensor to successfully navigate using a scene familiarity algorithm [18]. However, few reports have been published detailing scene familiarity based algorithm performance using natural scenes. In particular, questions still remain as to whether natural scenes, either indoor or outdoor, contain enough visual information to prevent scene aliasing using simple vision based scene familiarity algorithms.

In this paper, we examined the relationship between sensor parameters, visual information and success of route recapitulation in an indoor environment. We sought to implement the previous version of our scene familiarity algorithm ([18]) in a relatively feature-dense indoor setting and address whether successful recapitulation could be directly related to sensor resolution and degree of sensor image rotation. Additionally we asked whether the information in this setting was sufficiently distinct among views to prevent aliasing.

We generated a high-density visual survey of our laboratory and adjoining corridor, consisting of 2816 panoramic snapshot scenes 1.3 m above the floor. An information landscape map was produced based on a pixel-by-pixel comparison of all sampled scenes. We examined the translational and rotational image differences within the landscape using sensors at various resolutions. An analysis of several corridors was also carried out to investigate the visual information in homogeneous and repetitive settings. We produced a variation of our scene familiarity algorithm that tracks previously experienced routes and used it to test the influence of sensor parameters on recapitulation success. We also show that our navigation algorithm can successfully recapitulate complex paths, including a path with its end point obstructed from the path starting view. Finally, we found no scene aliasing between a sample of our corridor scenes and >25,000 other corridor, laboratory, and outdoor scenes.

## Methods

### Laboratory survey

**Fig 1:**
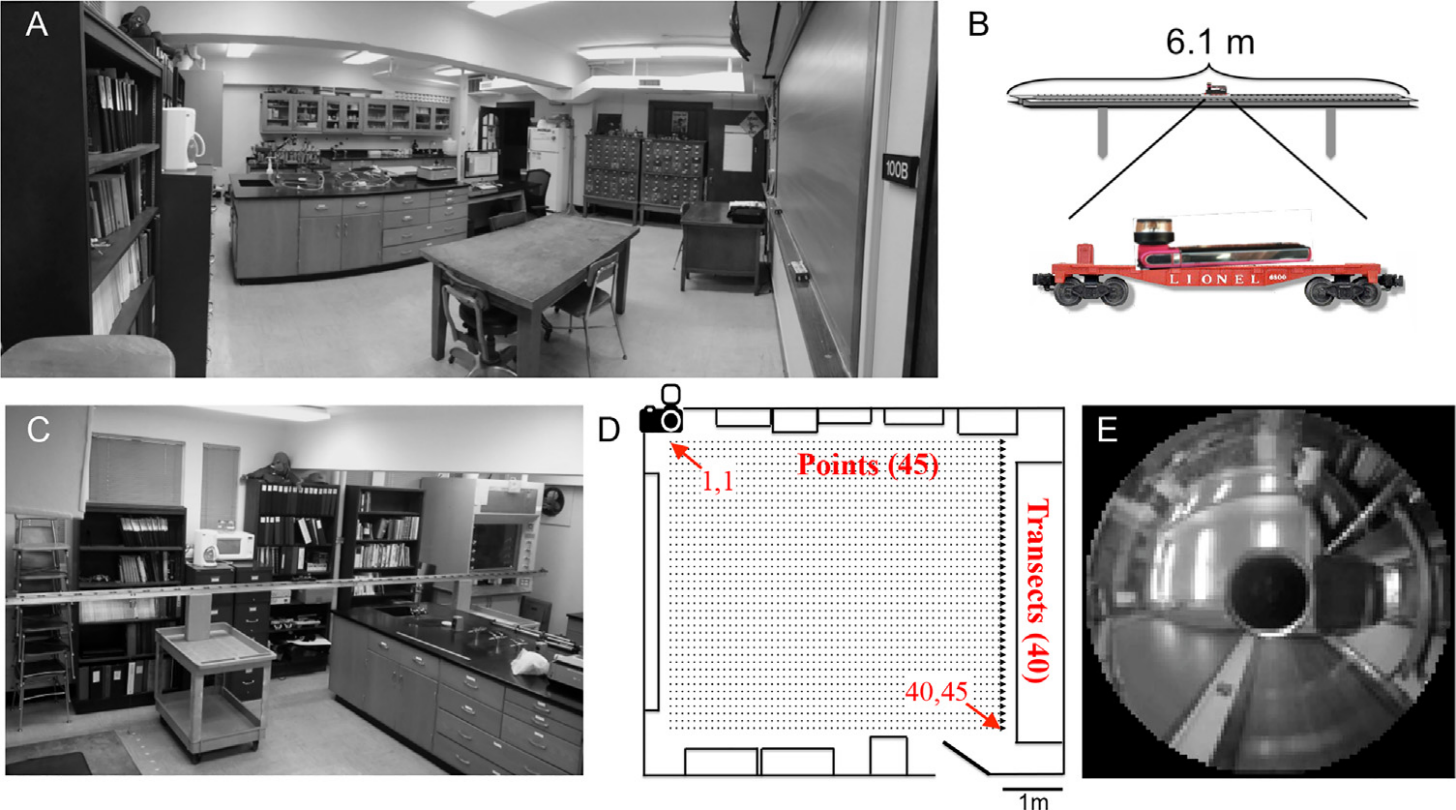
Scene acquisition. **A** Wide-angle view of the laboratory setting for this study, as viewed from the southeast corner. **B** A video camera with a 360° lens was mounted on a model train flatcar and placed on a track (**C**) resting upon cinder blocks on two movable carts. **D** The laboratory was divided into 40 transects formed by moving the track in ~12.7 cm intervals. For each transect, the flatcar was moved along the track and still images were taken every 12.7 cm, to create 45 images (“points”) per transect and a total of 1800 images for the room (40x45; scale bar = 1 m). **E** Sample circularized image with pixel resolution reduced to a 100x100 matrix.

Our laboratory at the University of Oklahoma was our main survey site. The room is a 7.3 m x 6.9 m rectangle with a 2.8 m ceiling height. Objects along the walls of the lab include a chalkboard, bookshelves, a fume hood, a refrigerator, card catalogs, laboratory countertops, storage cabinets, and three doors (Fig 1A). A built-in lab bench forms an island in the middle of the room. We custom built a camera support structure by abutting and fastening two 6.1 m aluminum C-channel beams (2.5 cm wide x 5.1 cm tall) along their middle to form a rigid I-shaped platform. Atop this platform we connected and affixed six lengths (each 101.5 cm) of O gauge model train track (Lionel, 6–65523) on which rode a model train flatcar (Lionel, 6808) to serve as our camera dolly (Fig 1B). On the flatcar we positioned a digital video camera equipped with a panoramic lens (Sony Bloggie, model MHS-PM5K).

We supported the track system on two cinder blocks positioned 1.5 m from the track ends. The cinder blocks rested on two standard laboratory carts to allow the track to be moved to desired positions across the room (Fig 1C). The track was oriented parallel with the long dimension of the room and raised to a height of 115 cm to clear the lab bench island; the final height of the camera lens was 128 cm. The train track crossties, spaced at every 12.7 cm (5 inches), were used as convenient intervals for image capture. Forty-five equally spaced images (points) across the length of the track were included in our visual landscape. Along the short dimension of the lab we made two parallel sets of markings on the floor, spaced at 12.7 cm, to serve as a guides for moving our transects across the room. We used plumb bobs on the track ends to guide the carts to the marked positions. We fitted 40 such transects across the room after allowing for the space occupied by the fume hood, refrigerator, shelves, etc. In the end, we generated 1800 equally spaced images in a 40 x 45 grid in the laboratory and considered this the image landscape of our lab (Fig 1D). We used MATLAB to crop each image to a square, enhance the image contrast with the histeq function, and pixelate to 100x100 and 10 levels of gray. We then circularized each image, turning all pixels on the outside of the circle black (Fig 1E). We stored these 1800 circularized images in a MATLAB structured array.

To generate an extra landscape outside the view of our laboratory landscape, we continued our survey through the lab entryway and into the corridor adjacent to our main laboratory. The image survey and landscape generation were done as detailed above. Therefore, the combined laboratory and corridor landscape included three extra sets of image grids: one 6 x 14 grid leading to the door entryway, one 3 x 6 grid over the door threshold, and one 20 x 45 grid in the corridor, for a total of 2816 (1800 + 98 + 18 + 900) images.

### Visual information analysis

We used the 1800 surveyed images at 100x100 pixel resolution to generate an information topographic map that depicts the overall quality of the laboratory room’s visual information. To do this, we summed the absolute pixel-by-pixel difference of each scene matrix compared to all other room scenes. We then calculated and plotted the average image difference values by scene position to generate a surface map based on visual familiarity within the room.

We also assessed the interplay of visual information and sensor resolution by analyzing translational and rotational image information for sensor resolutions ranging from 10x10 to 80x80. We assessed translational information by first calculating the pixel-by-pixel absolute scene differences (image difference function or IDF) of each scene relative to all other scenes, without any image rotation. We then normalized these values based on the maximal difference within each resolution and generated a set of volcano plots for each sensor resolution. We then made north-south, east-west slices through each plot and folded each at its focal scene minimum to align the falling and rising phases of each slice. This produced four rising curves for each of the 1800 volcanoes, or 7200 separate vectors for the entire room. Finally, we averaged these 7200 vectors with distance from the focal scene to produce a single curve for each sensor resolution. We assessed rotational information (rotational information difference function, or RIDF), by rotating each scene 360 degrees in 1-degree intervals and calculating pixel-by-pixel absolute scene differences of each rotation relative to its original orientation. We normalized each set of curves by the maximal differences within each resolution, aligned the falling and rising phases of each of the 1800 curves to generate 3600 vectors, and averaged these for each 1-degree rotation, again producing a single curve for each sensor resolution. Finally, for each sensor resolution, we calculated the half maximal value (p50) for the translational (meters) and rotational (degrees) information curves.

### Training paths and quiver plots

We produced a graphical user interface (GUI) in MATLAB to visualize the room and allow the user to manipulate the sensor resolution, number of visual angles surveyed, threshold for a familiarity match, and training path length. To create the training path, the user first enters the number of training path points to be placed and then moves cross-hairs across the room’s map to position each point. The program then generates a continuous training path by plotting the segments between contiguous points and selects the appropriate scenes intersected by the path from the 1800-scene image base. The program then uses a 360-degree rotation system analogous to a compass to calculate the bearing between successive user-placed points and rotates each scene by its segment’s bearing to form a set of images representing what the agent would experience as it moves along the training path. Thus our training path consisted of a sequence of images from our 1800-scene database all in the correct orientation as if an agent had just experienced them as a path.

We generated quiver plots to show both the degree of familiarity (arrow length) and the orientation (arrow bearing) of the best training scene match from any point in the room. Each quiver plot consists of 1800 red arrows with contours and shadings indicating isotropic regions of similarity relative to the training path scenes.

### Scene familiarity tracking algorithm

**Fig 2:**
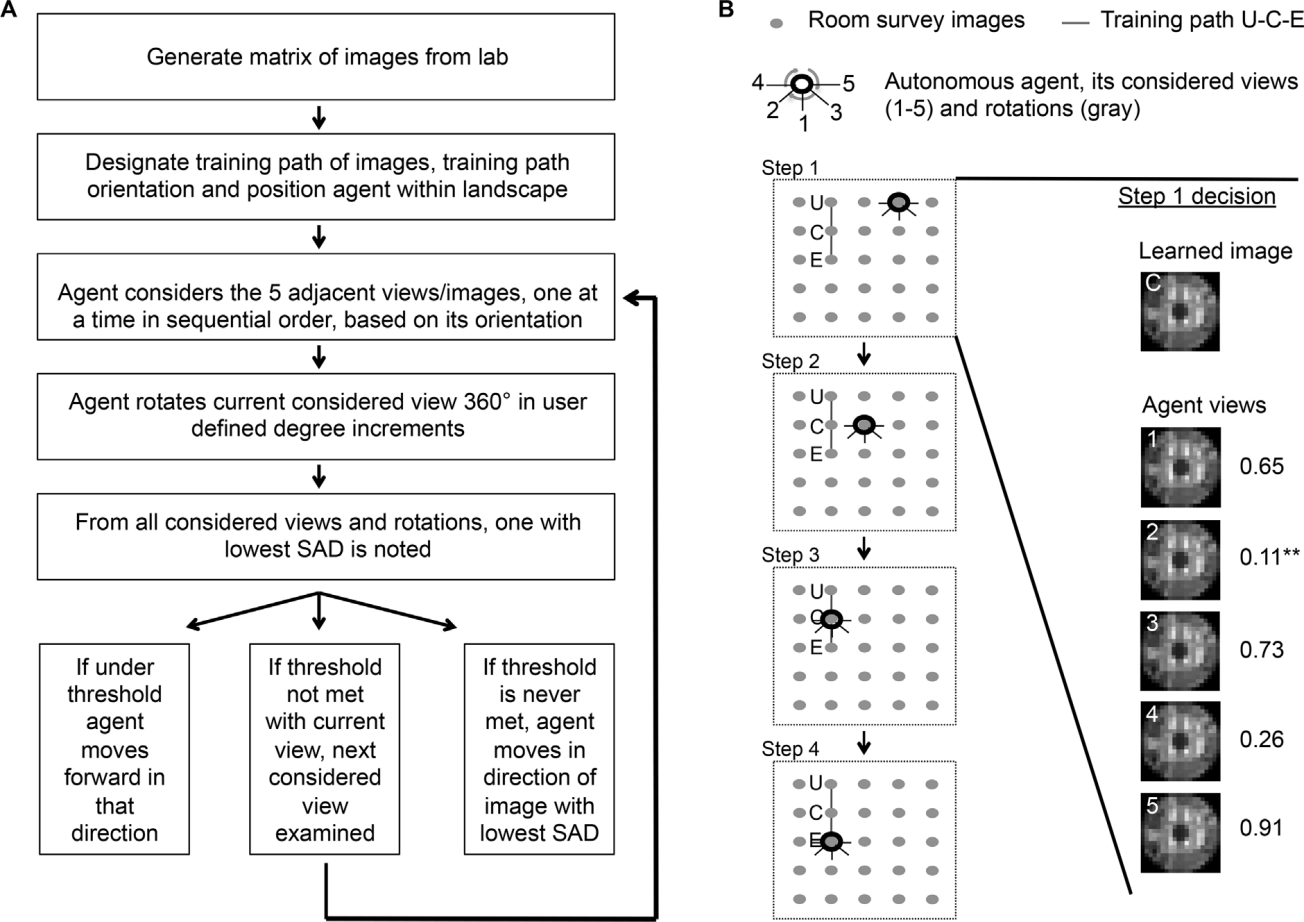
Schematic representation of the scene familiarity tracking algorithm. **A** Flowchart showing an overview of image acquisition and the workflow of the scene familiarity algorithm. **B** A simplified version of how the algorithm selects the direction it will move in the landscape. Each gray circle in the 5 x 5 array represents a captured image. The training path consists of three points, labeled U-C-E and linked with a gray line. A thick black ring represents the autonomous agent. Black lines indicating its considered views (1–5) are shown emanating outward. Gray lines around the ring indicate the agent’s rotational scanning capabilities. Steps 1–4 highlight a series of steps taken by the autonomous agent in a hypothetical example of path recapitulation within the landscape of surveyed images. In step 1, the agent is placed within the landscape two images to the right of training path image U. Step 2 shows the agent after analyzing its considered views, chooses view 2 as the lowest representative summed absolute image difference (SAD) and moves from its position in step 1 in the direction of image C. This decision is highlighted with a series of representative images. Shown is the designated training path image C and the 5 considered views as well as calculated SAD values for each. Step 3 and 4 shows the agent finding and then progressing to the end of the training path. Once at the end of the path, the agent is unable to progress, as there is no recognition of a direction for the next image. The agent therefore begins a series of short movements back and forth around image E.

We produced an algorithm to test if the room’s visual information is adequate for navigating a training path (Fig 2). Once a training path is established and stored as described above, the user sets a familiarity threshold of 2 or higher. The threshold value is used as a divisor of the average scene difference value between the surveyed and training path scenes. The higher the threshold, the better the match must be before the agent can move on. For example, if the threshold is set to 2, and the average scene difference value is 1000, then a familiarity value of 500 is required to allow the agent to move on; a threshold of 4 requires a familiarity value of 250 to allow the agent to move on. The agent is then positioned at a starting point within the landscape. The agent considers five adjacent views (captured landscape scenes) based on its current orientation, one at a time in the following sequence: 1) front, 2) 45° right, 3) 45° left, 4) 90° right, 5) 90° left. The agent rotates the considered view 360° in user-set intervals, comparing the summed absolute pixel-by-pixel difference (SAD) of each rotation to all scenes in the training set (without regard to order). The agent notes the bearing of the image with the smallest difference and, if below the user-set threshold it steps to the nearest image in that direction and repeats the process. The nature of the MATLAB algorithm is such that all rotations are considered simultaneously through the use of a 3D array containing all rotations of the considered scene and the “repmat” function to make a 3D array with clones of each training path scene to the same number of considered rotations. This way, the minimum SAD and corresponding angle are found instantaneously by comparing the two 3D arrays. After rotating, if the considered view does not fall below threshold, then subsequent considered views are analyzed until threshold is met. If threshold is never met, the agent moves in the direction of the image and rotation with the smallest absolute sum difference value and repeats the process.

### Simple and complex route recapitulation at various sensor and navigation parameters

We used a variety of training paths, ranging from simple to complex, to test route recapitulation success using our scene familiarity algorithm. Within our GUI, we constructed a simple path with two smooth curves. The agent was then placed at 56 spots within the room, and recapitulation success (reaching the end of the training path) was assessed for each placement. The recapitulation success was further analyzed at sensor resolutions of 10x10, 20x20, 40x40, and 80x80. Each test was carried out at 10 gray levels, at 1-degree angle rotations, and with a familiarity threshold of 4.

Recapitulation success of a complex “ou” path within our laboratory landscape was assessed for a variety of sensor resolutions, rotational intervals, and familiarity thresholds. Time (T) and cumulative distance (D) covered during recapitulation was also assessed for each successful run. “T” was defined as the total number of scenes considered by the agent. Since considering multiple scenes adds time to the actual recapitulation, this was used as a proxy for the amount of time required for successful recapitulation. “D” was calculated by summing the distances of the selected scenes used for recapitulation to the nearest training path scenes.

### Path recapitulation of a novel path with our scene familiarity algorithm

We set the train car and Bloggie camera on a wheeled cart (at the same height as the surveyed scenes) and recorded video as the cart was pushed through the laboratory in a “U” shaped path. We extracted a set of 50 frames from the video and constructed a training path in our MATLAB GUI as described above. The agent was placed near the start of the training path and recapitulation of the path by the agent was determined using the visual landscape images of our laboratory. We used a modified version of our algorithm that only considers views 1–3 (front, 45° right, and 45° left). We used a sensor resolution of 40x40, 10 gray levels pixel depth, and 360 angles of inspection to generate these plots.

### Visual information analysis of corridor training paths

We analyzed the visual information in 12 additional locations on the campus of the University of Oklahoma. The locations were chosen based on their relative homogeneity and/or repetitive nature. Nine locations were building corridors, two were concrete tunnels between campus buildings, and one was an outdoor walkway. We assembled the Bloggie camera on a wheeled cart at a height of 128 cm from the floor, in a manner similar to that described above for the novel path. Nothing was disturbed prior to or during the filming of each video. The wheeled cart was pushed along the center of the corridor while preventing any human interference with the path. Recording was carried out within each corridor in both directions. We used a 30 Hz video frame rate and captured fifty evenly spaced scenes for the analysis of visual information within each corridor. We used all of the video frames (>25,000) to assess visual difference information relative to the 900-scene image survey of the corridor outside our laboratory.

## Results

### Visual information analysis

**Fig 3:**
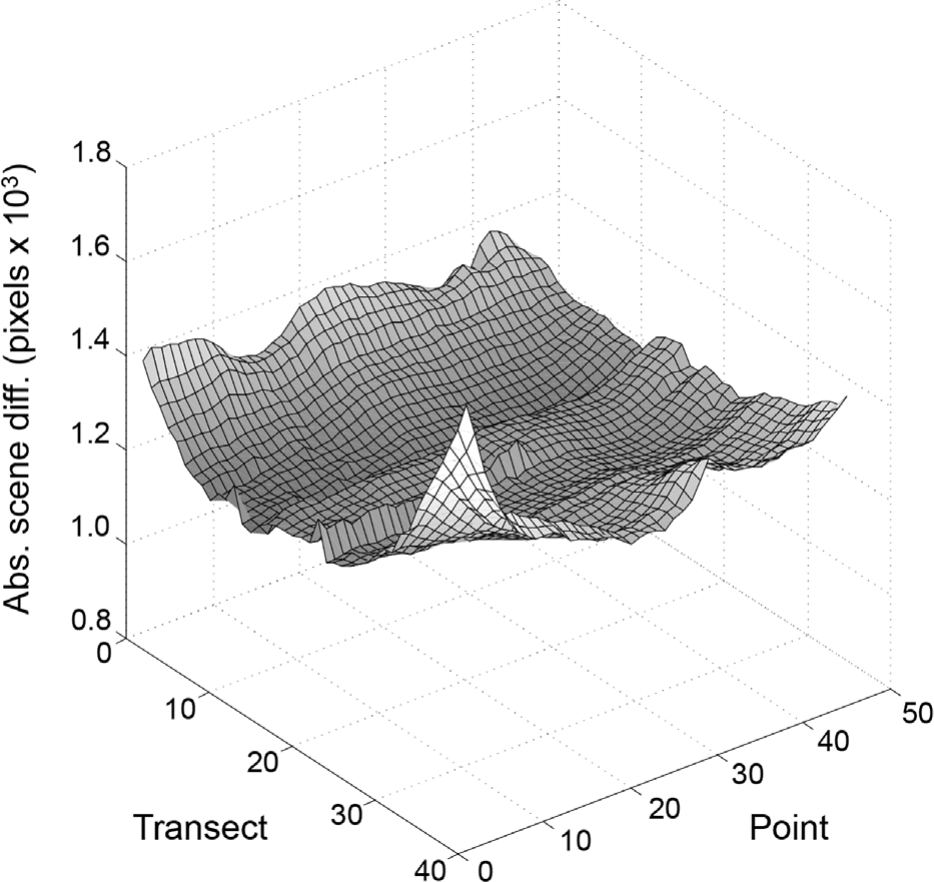
Scene familiarity landscape. Each scene was processed to a circularized 100x100 pixel, 10 gray level matrix and compared to all 1800 scenes, including itself. The surface plot indicates average absolute scene difference (Abs. scene diff.) values by scene position.

The comparison of individual scenes in our laboratory revealed a high amount of visual information within the lab. This is important because the higher the amount of distinct visual information the more likely route recapitulation success will be achieved. To address the room’s visual information, we generated a scene familiarity landscape by calculating the mean of the SAD for every surveyed image in the room against every other image for a sensor resolution of 100x100 (10 levels of gray). This landscape (Fig 3) forms a shallow trough with the lowest difference values (~1.2 x 10^4^ pixels) near the center of the room and higher values near the north and south walls. The greatest average difference (~1.7 x 10^4^ pixels) is at point 1 on transect 40, which is next to a door that opens to an adjacent room. The information differences near the east and west ends are lower than near the north and south walls because of homogeneity introduced by a large chalkboard on the east wall and a long bank of lab cabinets on the west wall. The small ridge along transects 22–23 is attributed to a thin (~3.8 x 3.2 cm) electrical cord channel that extends from the lab bench island to the ceiling, forming a slender pillar in the middle of the room.

**Fig 4:**
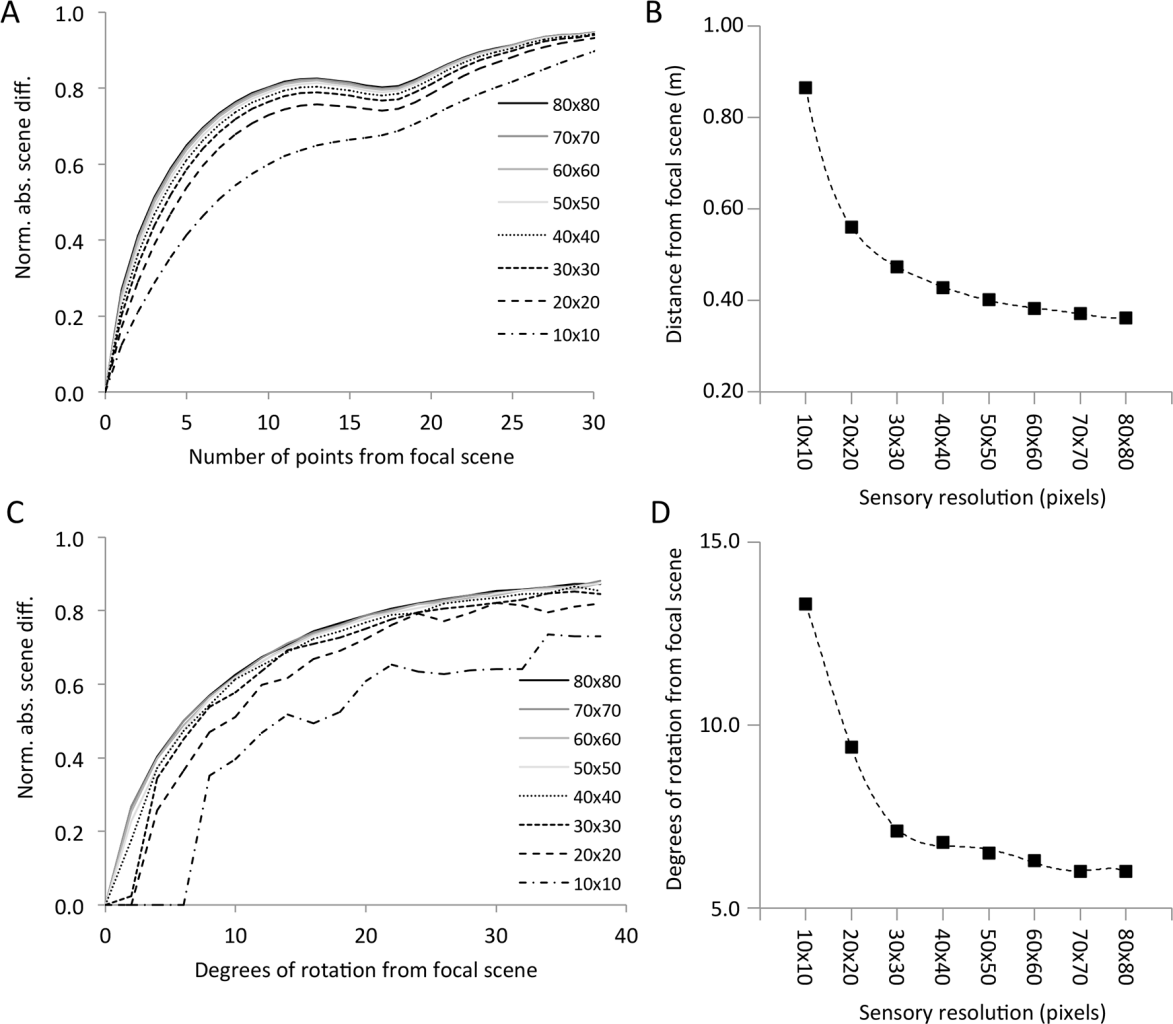
Average IDF and RIDF plots. **A** We produced volcano plots comparing normalized scene differences (IDF) of each scene to all 1800 scenes in the room. Slices were taken through each plot along the north-south and east-west axes. Each slice was then divided at its focal point, with the falling phase of each slice inverted and aligned with the rising phase. This produced four rising curves for each of the 1800 volcanoes, or 7200 separate vectors for the entire room. The family of curves shows average values of these 7200 vectors with distance from the focal scene for each of the eight sensor resolutions (all at 10 levels of gray). **B** Plot of p50 values in meters (= points x 12.7 m) from the focal scene for the eight sampled resolutions. Dotted line is a 6^th^-order polynomial fitted to the points (R^2^ = 0.99996). **C** RIDF plots were produced for all 1800 scenes at 360 degrees for the eight sampled sensor resolutions (all at 10 levels of gray). Only the first 40 degrees are shown because the curves remain flat to 180 degrees. The plots were produced as described in A, except that the total number of vectors was 3600. **D** Plot of p50 values in degrees for the eight sampled resolutions. Dotted line is a 6^th^-order polynomial fitted to the points (R^2^ = 0.99932).

Our work using images of outdoor settings has suggested that the success of learned path recapitulation is related to visual information and sensor resolution [18], which is in line with other reports from both outdoor and indoor settings [15, 19, 23]. However, little information concerning this interplay previously existed from natural settings. Therefore, we sought to assess the visual information of the laboratory from various sensor architectures. We examined the visual catchment areas derived from scene comparisons from two perspectives: 1) IDF, or information differences with translational movement away from a focal view, and 2) RIDF, or information differences of each view rotated in 1-degree intervals away from its original (0 degrees) orientation. We calculated normalized IDFs and RIDFs (Fig 4A and 4C) for sensor resolutions ranging from 10x10 (100 pixels) to 80x80 (6400 pixels). We also calculated half-maximal (p50) values for normalized IDFs and RIDFs (Fig 4B and 4D).

The IDF (translational information) catchment areas are greatest for the lowest sensor resolutions (10x10, 100 pixels, p50: 0.86 m; 20x20, 400 pixels, p50: 0.56 m) and exhibit hyperbolic decay with higher pixel densities (80x80, 6400 pixels, p50: 0.36 m). The systematic dip in the IDF curves around 15–20 points from the focal view is caused by the disproportionate number of values used in the mean IDF calculation for points nearer the walls and the greater difference values for the north-south slices compared to the east-west slices. The RIDF (rotational information) catchment areas show similar trends. The catchment areas are greatest for the low pixel density sensors (10x10, 100 pixels, p50: 13.3 degrees; 20x20, 400 pixels, p50: 9.4 degrees) and constrict precipitously with higher densities (80x80, 6400 pixels, p50: 6.0 degrees). While the catchment areas are wider for lower density sensors, the curves are not as smooth, which may be partly due to nearest neighbor pixel interpolation of the MATLAB “imrotate” function that can cause some distortion of low-resolution images.

### Navigation by scene familiarity

**Fig 5:**
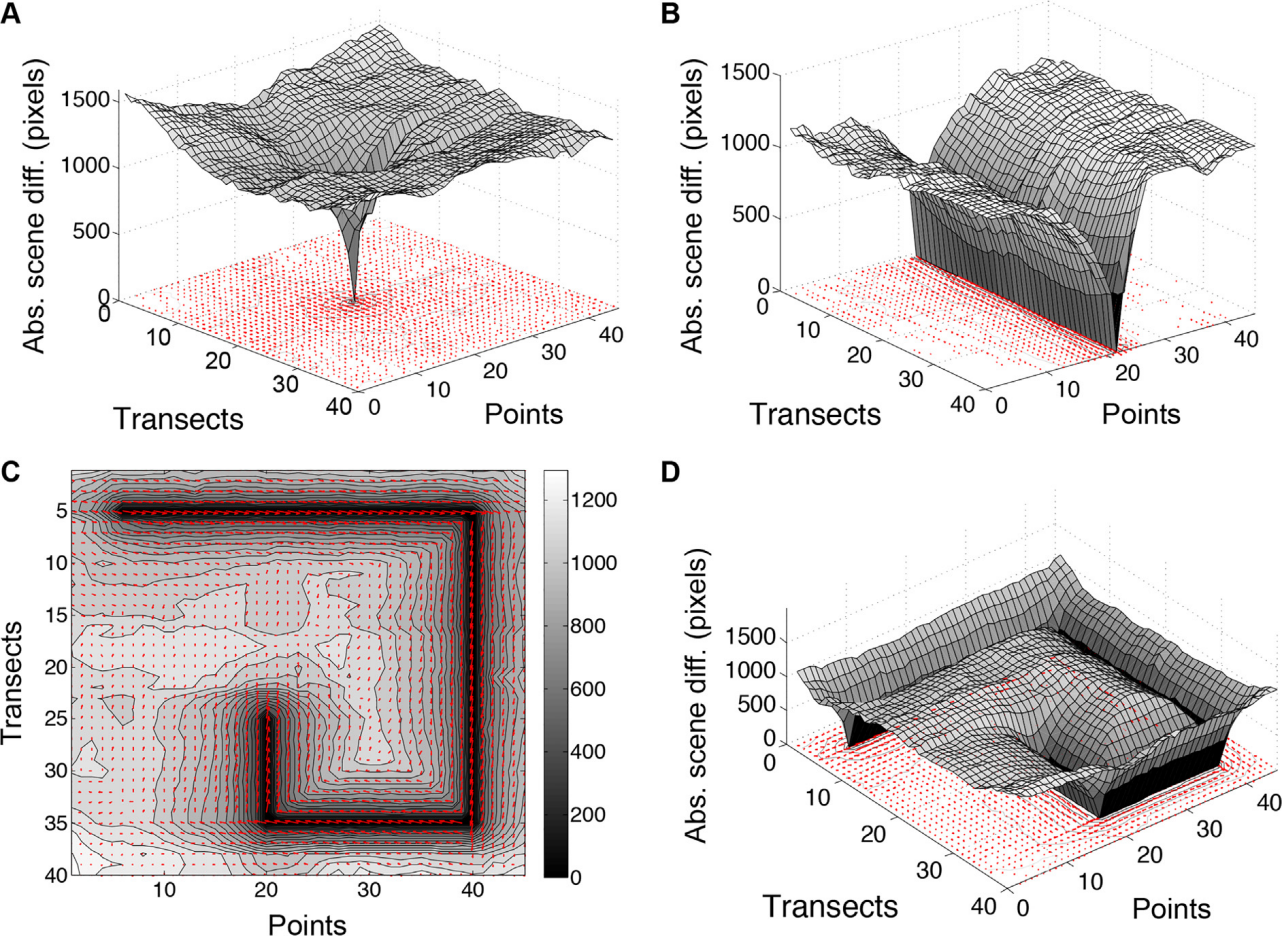
Scene difference plots produce volcanoes and troughs relative to focal scenes and paths. **A** The absolute pixel-by-pixel difference (Abs. scene diff.) of the scene at point 22 on transect 20 is plotted against all scenes in the room, at a sensor resolution of 100x100. **B** Surface plot of image differences compared to a straight line training path running from south to north across the transects at point 20. A contour plot [**C**] and a surface plot [**D**] of image differences are shown relative to a training path with three turns. In all images, darker shades indicate no or very small absolute scene differences, and lighter shades indicate larger differences. The red arrows indicate the optimal direction at each point as calculated by the RIDF.

Contour, surface, and quiver plots are useful for assessing information available for navigation via scene familiarity. Fig 5 shows a sample plot of scene differences between a single reference scene and all other scenes in the landscape (5A), a series of scenes comprising a straight line (5B), and a series comprising a three-turn (5C,D) training path. The SAD comparisons of a single point to the rest of the room produces a funnel-shaped surface plot, with the catchment area centered on the focal view and slopes that rise steeply with distance. The scenes with the lowest or minimum scene difference compared to the reference point are clearly found in the few scenes adjacent to the reference point. Similarly, troughs are formed when paths of surveyed images are selected and compared to the rest of the room’s images (5B, C, and D). The red arrows in Fig 5 indicate the optimal direction at each point as calculated by RIDF. As shown in the contour plot of Fig 5C, the directional arrows closely match the direction of the training path, which began at point 5 on transect 5 and made three turns before ending at point 20 on transect 25.

**Fig 6:**
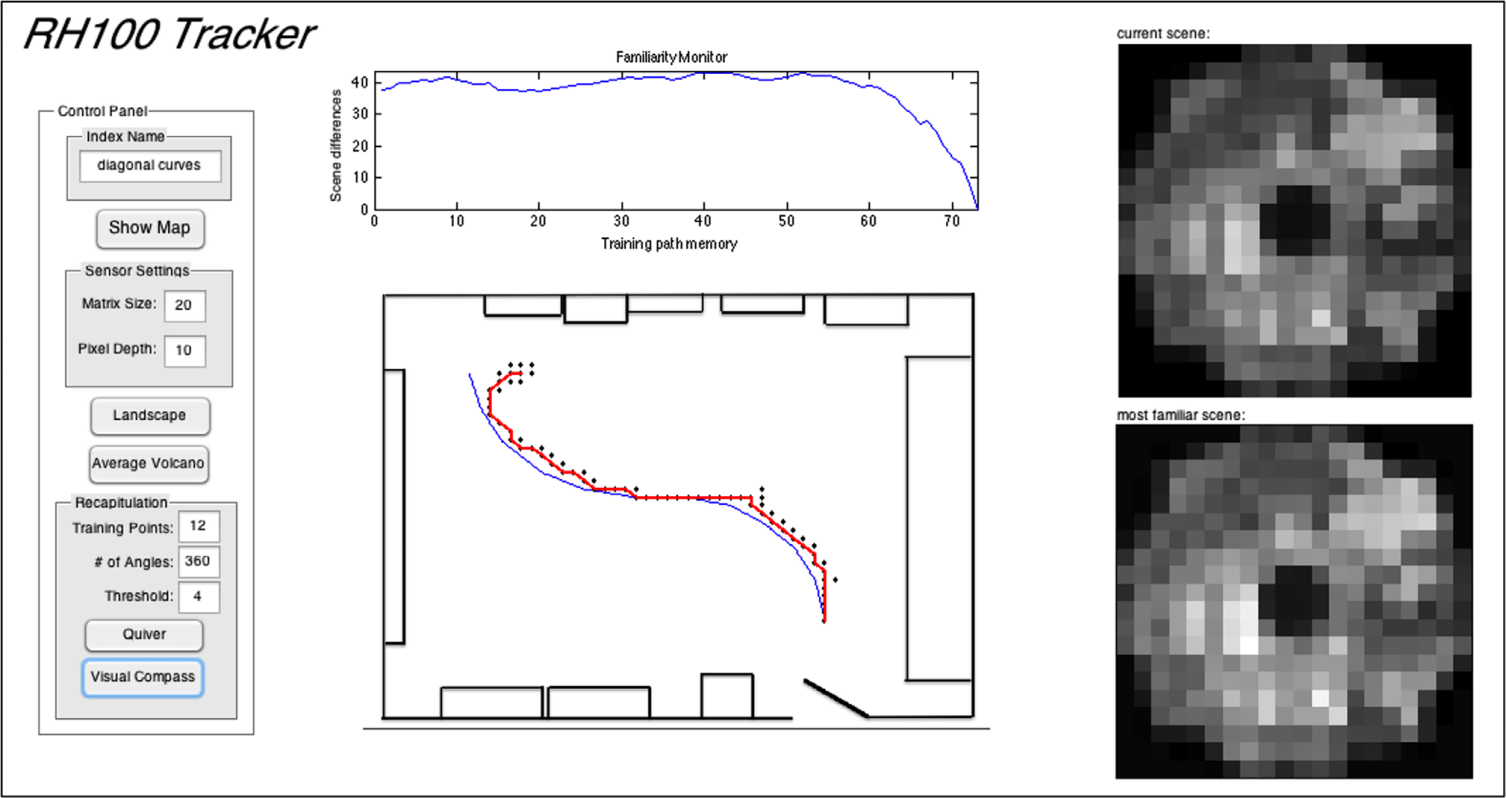
Tracking GUI and sample performance on a simple training path. The GUI contains a control panel (left) for user-set parameters; a map of the room (bottom center) with training path (blue line), surveyed scenes (black dots) and recapitulated path (red line); a familiarity monitor (top center) showing the IDF of the currently inspected scene to all scenes in the training path; and circularized, pixelated views (right) of the current scene and the most familiar training path scene.

**Fig 7:**
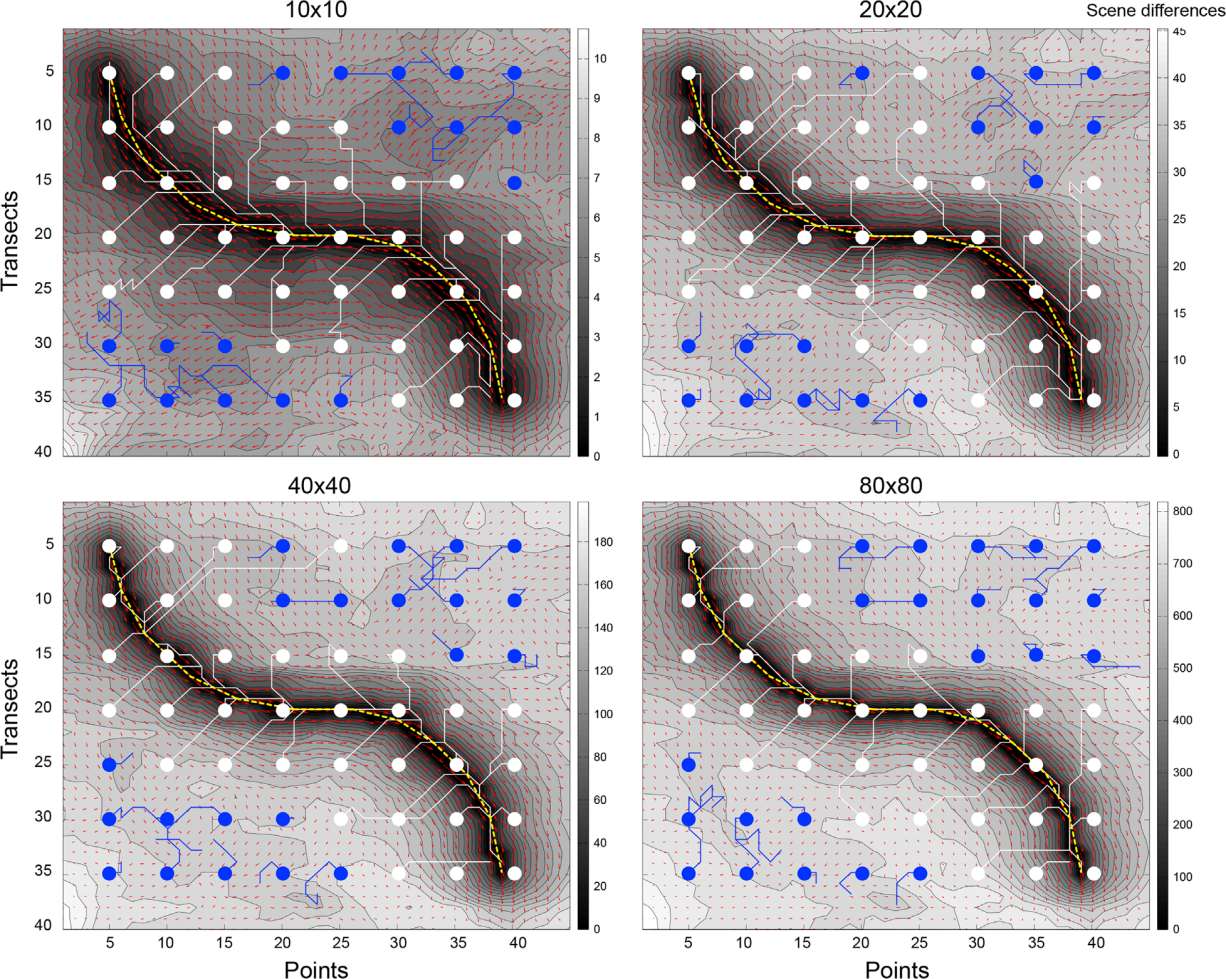
Performance of auto-tracking algorithm on simple training path using different sensor resolutions and starting points. Recapitulation performance along a simple training path with two smooth curves (yellow dashed line) is shown for four sensor resolutions: 10x10, 20x20, 40x40, and 80x80 (all were tested with 10 gray levels, 360 angles inspected, and a familiarity threshold of 4). Performance was tested for 56 equally spaced starting points. Circles indicate starting points, and lines are agent routes. White circles and lines indicate successful recapitulations (within 2 points of the end of the training path); blue circles and lines show unsuccessful attempts. The routes are superimposed on contour and quiver plots generated for the relevant sensor architectures.

Our GUI eased the manipulation of attributes such as the sensor resolution, the number of considered angles, and the familiarity threshold during recapitulation. Fig 6 shows the GUI during the recapitulation of a simple training path consisting of two curves (the path used in the trials of Fig 7). The control panel at left allows indexing, user control of matrix size and pixel depth, choice of analyses (familiarity landscape or point-wise volcanoes), training path input, and recapitulation parameters and displays. The lower portion of the center panel has a diagram of the room with the training path shown in blue. The black dots are the considered views used by the agent during a recapitulation run; the red line connects the final selected views. The familiarity monitor at the top of the center panel indicates the degree of similarity of the currently considered view to all sequential views making up the training path (in this case about 75). The right panel shows the circularized, pixelated view of the current view on top and the best matched training path view below. In the trial shown in Fig 6, the agent started to the right of the beginning of the training path, made its way to the path, and then followed the path to the end (with some minor departures at the curves).

The IDF analyses of Fig 4 suggest that the catchment area of familiarity around a training path should widen with lower sensor resolution, while the precision of route-following should increase directly with resolution. We therefore expect that a displaced agent with a low sensor resolution would be able to return to the training path’s catchment area from a farther distance as compared to an agent with a higher resolution sensor. On the other hand, we also expect that an agent with a higher sensor resolution should track closer to the training path than an agent with lower sensor resolution. Fig 7 shows the tracking performance of an agent along the same training path from 56 different points in the room, at four different sensor resolutions (10x10, 20x20, 40x40, 80x80). Dashed yellow lines represent the training path. White and blue circles and lines indicate starting points and agent paths, respectively. White indicates a successful recapitulation; blue indicates an unsuccessful attempt. These paths are superimposed on contour and quiver plots (see Fig 5C). The lower density sensors had more successful recapitulations from points farther from the training path (10x10: 39/56; 20x20: 40/56) compared to the higher density sensors (40x40: 35/56; 80x80: 34/56). Furthermore, the successfully recapitulated paths tracked closer to the training path as resolution increases.

**Fig 8:**
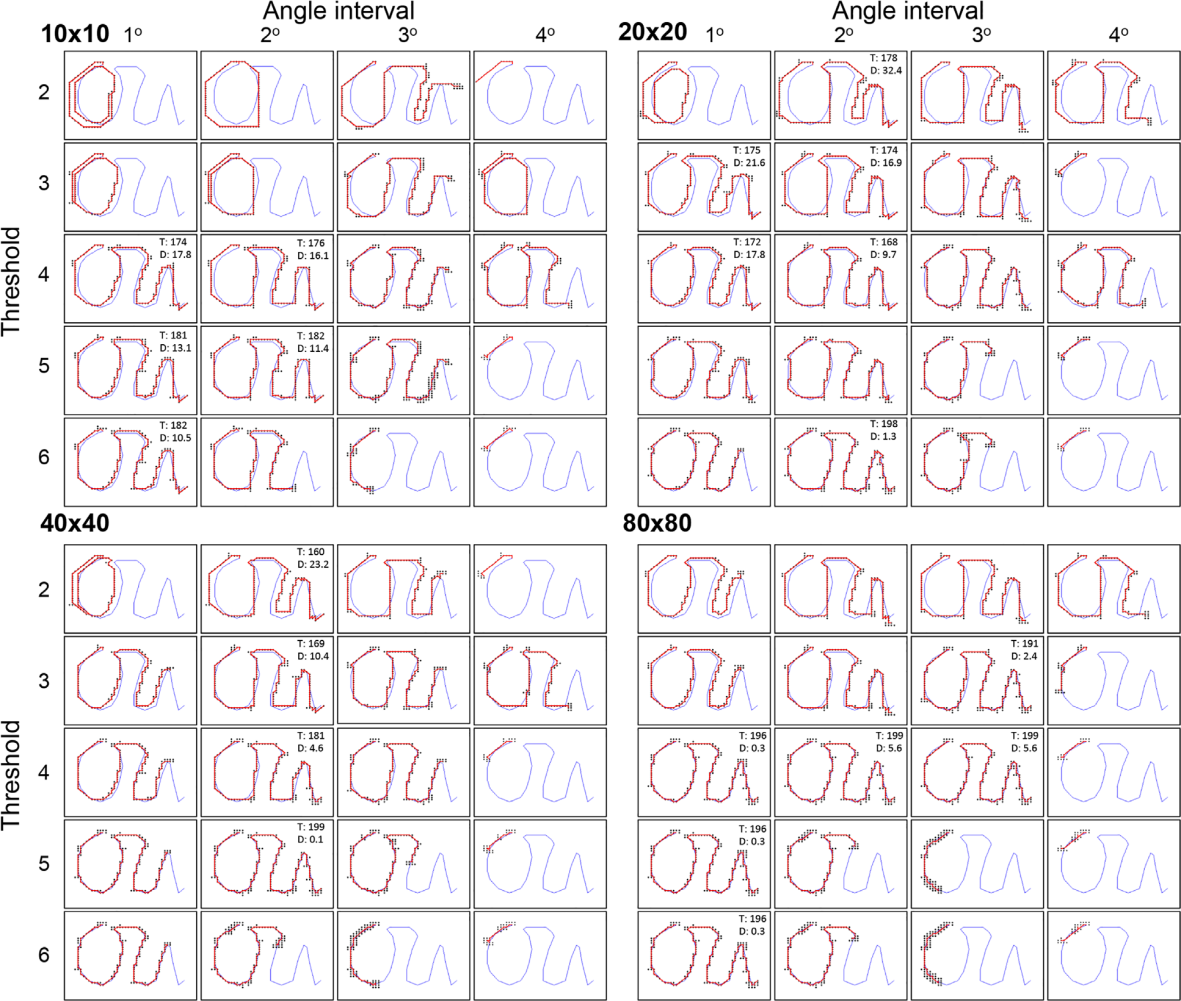
Performance of algorithm on complex training path. We generated a complex training path that roughly formed a cursive script of the lower case letters “o” and “u.” We tested the auto-tracking algorithm on this path while varying the sensor matrix density (10x10, 20x20, 40x40, 80x80), the interval between angles surveyed, and the familiarity threshold (blue line = training path; red line = agent path; black dots = scenes surveyed). The insets show the number of scenes surveyed (T) and the total distance of departure between the red and blue lines (D) for the trials that made it to within two points of the end of the training path. T is a proxy for the time it took to complete each successful recapitulation, and D is based on the cumulative distance (in meters) of departure from the nearest training path points.

Next, we examined the ability of our agent to recapitulate a complex training route while varying sensor density, number of angles surveyed, and familiarity threshold. Fig 8 shows the results for four densities (10x10, 20x20, 40x40, 80x80), four rotation degree intervals (1°, 360 angles; 2°, 180 angles; 3°, 120 angles; 4°, 90 angles), and five familiarity threshold levels (from 2 to 6). We assessed successful path completions and both the number of scenes surveyed (a proxy for time) and total departure of the recapitulated path (shown in red) from the training path (shown in blue). Successful recapitulations depended on all three parameters. For example, no agent was able to complete the recapitulation with the angle interval set to 4 degrees, no matter the resolution or threshold. The number of successful trials varied with resolution (five for 10x10; six for 20x20; four for 40x40; six for 80x80). In general, agents with the lower resolution sensors completed their courses faster (fewer surveyed points) but had greater total departures compared to those with higher resolutions. Also, the wide catchment areas for the lowest resolution (10x10) produced more aliasing in the form of erroneous loops around the “O” part of the training path (five for 10x10 compared to one for both 20x20 and 40x40, and zero for 80x80). See S1 Video through S5 Video in the supplemental information for examples of complex route recapitulations from our GUI. We think that some of the unsuccessful recapitulations were due to the agent getting “stuck” while stepping back and forth between scenes. The algorithm saved training path scenes as 360-degree panoramic images, and the recapitulation routine examined a full rotation of considered scenes. These errors could be reduced by constraining the agent’s field of view during both training and recapitulation routines.

**Fig 9:**
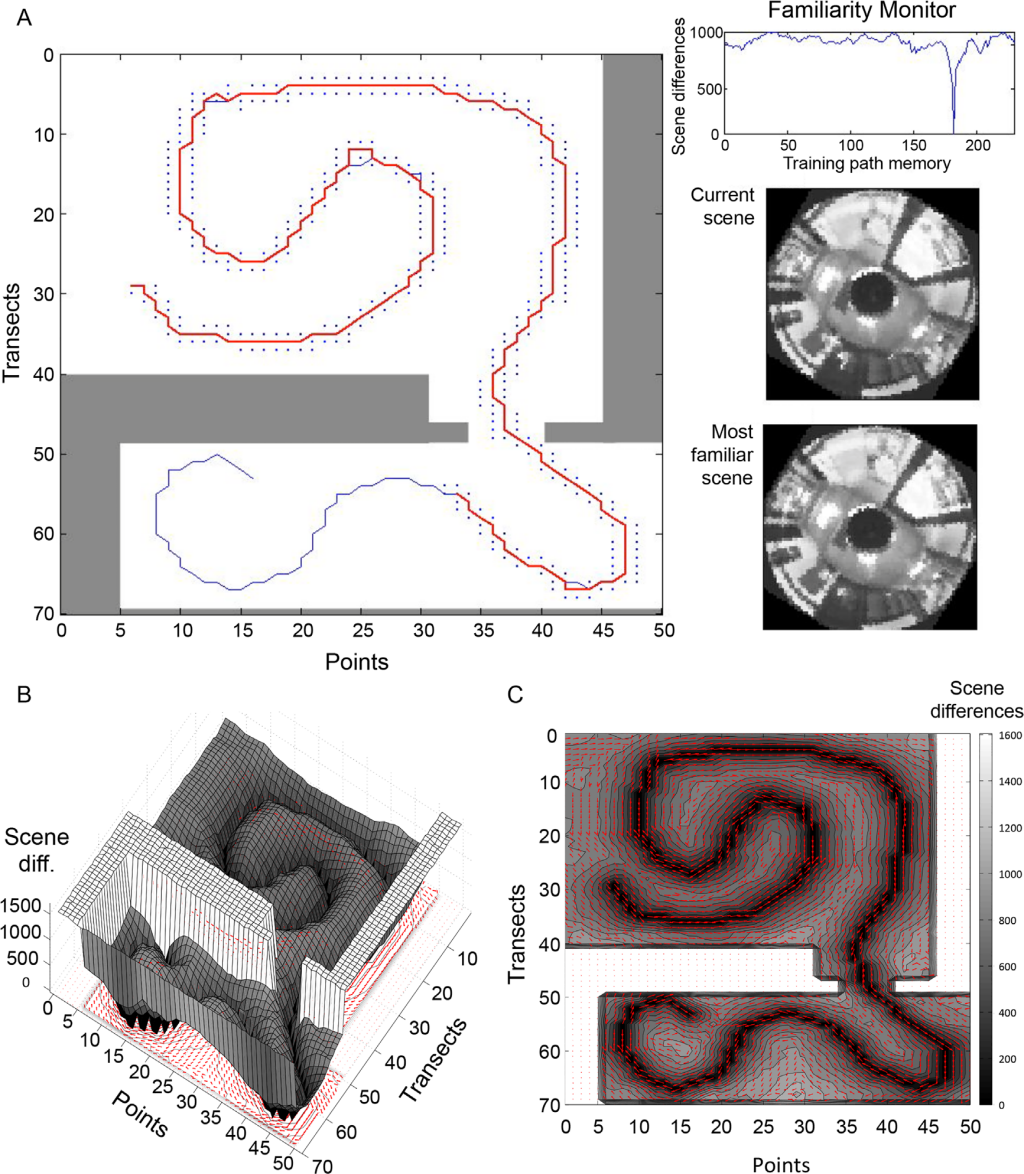
Auto-tracking from lab to adjacent hallway. **A** The visual landscape was extended through the doorway and into the adjacent hallway using the same train track system described in Fig 1. A complex training path (blue line) started near transect 30, point 5 in the laboratory and made a looping path through the room, doorway, and hallway before ending near transect 55, point 15. The red line is the recapitulated path (in progress) and the blue dots are the previously inspected points. The familiarity monitor at top right shows absolute scene differences of the currently inspected scene to all scenes in the training path memory and the images below show the current scene and the most familiar scene in memory, both pixelated at 80x80. **B** Surface plots and (**C**) contour plots of the absolute scene differences in the room and hall relative to the training path show distinct catchment valleys of image similarity along the entire training route. Red arrows (quiver plots) below each plot indicate the best angle of rotation (arrow direction) and strength of image match (arrow length) at each point.

Bees and ants can navigate long distances from the hive or nest to a feeding site, which is often out of view when leaving the nest. To test the ability of our algorithm to successfully recapitulate such a path, we extended our visual landscape to include images through the laboratory doorway and a portion of the adjacent corridor. A complex meandering training path was created that began in our laboratory and traveled through the doorway and into the corridor, with the end point obstructed from the start point by a wall (Fig 9). Accurate route recapitulation was achieved with very little deviation from the training path (Fig 9A). The surface and quiver plots (Fig 9B and 9C) show distinct catchment areas along all parts of the path. The familiarity monitor also clearly shows the steep increase in image difference between the current scene and all other scenes from the visual landscape, even as the point is obstructed from the path starting view. (S6 Video in the supplemental information shows a complete path recapitulation and S7 Video shows a fly-through of the image difference landscape of a similar path recapitulation.)

**Fig 10:**
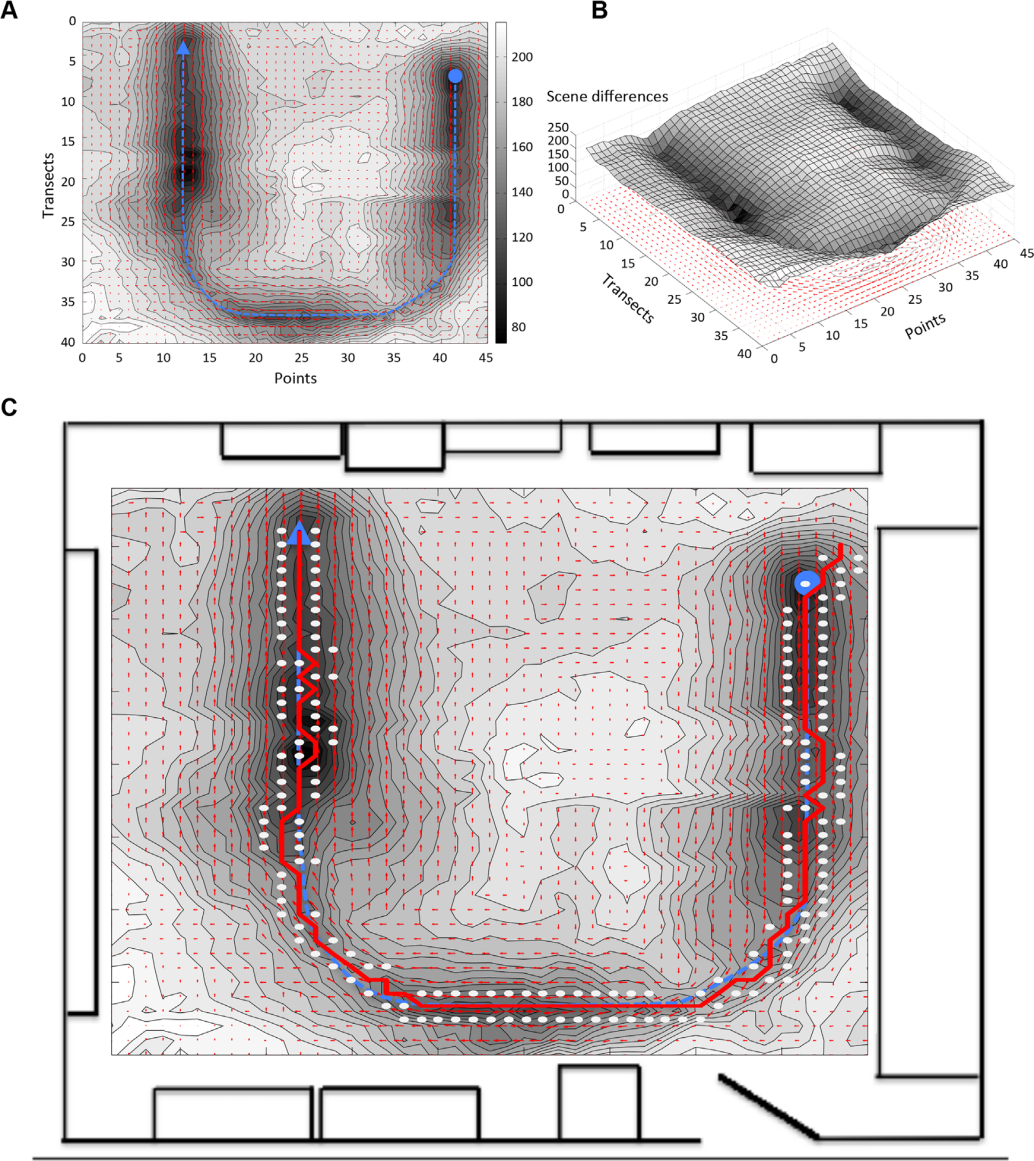
Performance of algorithm relative to “real” training path. The Bloggie camera with panoramic lens was mounted on a lab cart at the same height as the snapshot landscape. We maneuvered the cart in a U-shaped training path starting near the fume hood (near transect 6, point 42), passing around the laboratory island, and ending at a bookshelf (near transect 2, point 12). A contour and quiver plot (**A**) is shown with the approximate location of the training path indicated by blue dashed line; a surface plot (**B**) is also shown for the same training path. We used a sensor resolution of 40x40, 10 gray levels pixel depth, and 360 angles of inspection to generate these plots. **C** The scene familiarity algorithm was used to recapitulate the path by identifying the best-matched scene (rotated to the best-matched angle) from the snapshot landscape to the scenes acquired during the training path video (white dots = surveyed scenes; red line = recapitulated path).

Our path recapitulation so far was carried out on training paths consisting of surveyed images linked together; we also tested whether a training path consisting of novel images could be recapitulated. We therefore produced a novel training path by maneuvering the Bloggie camera on a separate cart around the lab at the same height of the surveyed landscape. This occurred approximately eight months after the time the image landscape of the room was acquired. We extracted 50 scenes from this training path video and produced contour, surface, and quiver plots (Fig 10A and 10B) to show the familiarity information of the room’s scenes relative to this set of training path scenes. We then used a modified version of the auto-tracking algorithm to successfully recapitulate the path; Fig 10C shows a successful path recapitulation using a sensor pixel density of 40x40. In the previous path recapitulation, the auto-tracking algorithm considered 5 adjacent views in the landscape (one at a time) when deciding which direction to move. However, for the novel training path image set, the auto-tracking algorithm was modified to only consider views 1–3 (Fig 2B) and the best rotational match. Route recapitulation was successful using sensor resolutions from 10x10 to 100x100 (all were run using a 1° rotation interval). The sensors with pixel densities of 20x20 and greater accurately recapitulated the path with only a few minor departures in the turns. The 10x10 sensor, while still finishing the path, experienced more errors in choosing the correct image and direction and thus spent more time off the learned path; it navigated far off the learned path on the second turn, but eventually regained the catchment area and made its way to the final target. The 5x5 sensor navigated the first leg of the training route, but was unable to make the first turn in the “U” path. See S8 Video through S12 Video in the supplemental information for examples of recapitulations of the novel training path in Fig 10 at sensor resolutions from 5x5 to 100x100.

**Fig 11:**
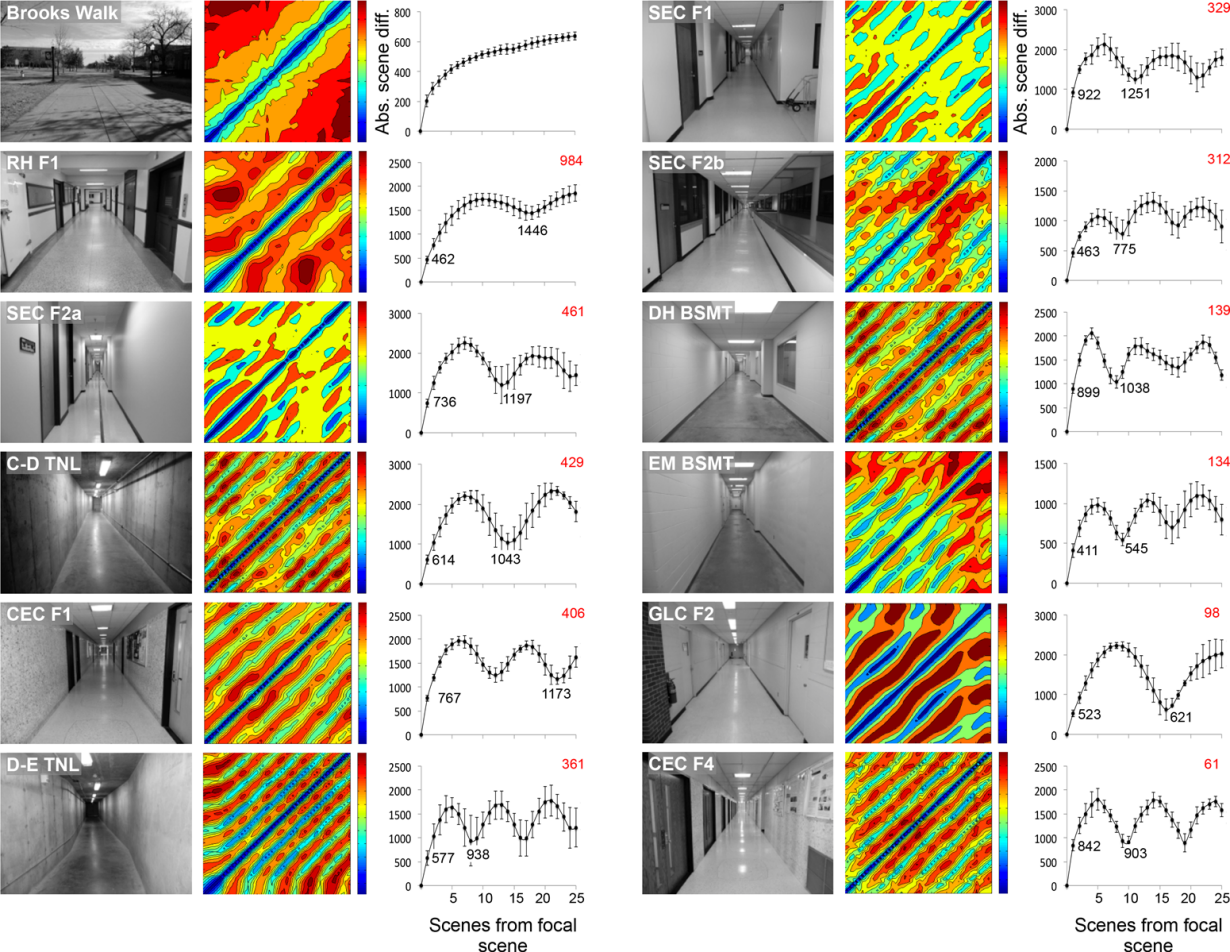
Corridor scene analysis. Panoramic videos along the length of 11 building corridors and one campus walkway were obtained with the Bloggie camera mounted at the same height (128 cm) as in the laboratory and hall surveys. For each corridor or walkway, a single video was made by pushing the cart with the Bloggie mounted in a straight line path down the center of the corridor or walkway at a constant speed. We used MATLAB to extract 50 evenly spaced scenes from each video, convert the scenes to 100 levels of gray, enhance the image contrast with the histeq function, and circularize and pixelate each scene to 100x100. The contour plots next to the still image of each corridor show cross-correlations of absolute scene difference values (with colors from blue to red indicating increasing scene difference values). The line graphs are plots of the mean SAD (± SD) for the nearest 25 scenes to each scene. We developed a rough index of the probability of aliasing by subtracting the SAD value of the nearest scene to the focal scene from the lowest local minimum within the set. These indices are shown in red in the upper right of each graph, with the highest number representing the lowest probability of aliasing. The corridors are arranged in descending order of this index. The outside walkway video produced no local minima and was deemed the most visually rich environment.

The visual information within our laboratory and adjacent corridor are sufficient for path recapitulation. Other indoor settings can be homogeneous, however, potentially causing aliasing and failure to successfully recapitulate some paths. We selected 11 corridors and one outdoor walkway, described in Table 1. We assessed the visual information within these settings by comparing the information of individual scenes to all other scenes in the same setting. Fig 11 shows the SAD values from a cross-correlation of each scene (n = 50) with all other scenes from that setting. Since a single linear path was made, the sequence of path scenes can be followed from the bottom to top and from left to right. For each corridor, scene difference values rise steeply with distance from the focal scene, as shown by the narrow blue-to-green transition in Fig 11 (contour plots). This is similar to what we observed in Fig 5 for our laboratory scenes. Additionally, the scenes with the nearest SAD value are neighboring scenes along the path (before or after the focal scene in the path), as shown by the darkest blue values being near the focal scene. It is also clear from the corridor scenes that there are multiple scenes along the path that are similar to the focal scene, as indicated by the striped pattern of lower scene difference values (greens and blues, Fig 11, contour plots). When the mean SAD (+/- SD) value of the nearest 25 scenes was analyzed, local minima (dips in absolute scene difference) are observed for all of the corridors (Fig 11, line graphs). This indicates areas in the corridors where visual information is similar among the scenes. However, these local minima never equal the focal scene information for any of the corridors tested. The outdoor corridor tested (Brooks walk) had a steep rise in scene differences and slowly plateaued with distance from the focal scene and did not show any local minima. We also generated a rough index of the likelihood of scene aliasing by calculating the difference of the scene closest to the focal scene and the lowest local minima within the 25 nearest scene set (values in red, Fig 11).

**Table 1:** The corridor and outdoor videos used for scene aliasing analysis.

Setting name	Description	Campus building
Brooks Walk	Outdoor walkway	NA
Brooks walk	Outdoor walkway	N/A
RH F1	Corridor	Richards Hall
SEC F2a	Corridor	Sarkeys Energy Center, 2^nd^ floor a
SEC F2	Corridor	Sarkeys Energy Center, 2^nd^ floor
SEC F1	Corridor	Sarkeys Energy Center, 2^nd^ floor
CEC F1	Corridor	Carson Energy Center, 1^st^ floor
CEC F4	Corridor	Carson Energy Center, 4^th^ floor
DH BSMT	Corridor	Devon Hall, basement floor
C-D TNL	Concrete tunnel	Carson Energy Center to Devon Hall underground tunnel
EM BSMT	Corridor	ExxonMobil/Lawrence G. Rawl Engineering Practice Facility
D-E TNL	Concrete tunnel	Devon Hall to ExxonMobil underground tunnel
GLC	Corridor	George Lynn Cross Hall, 2^nd^ floor

**Fig 12:**
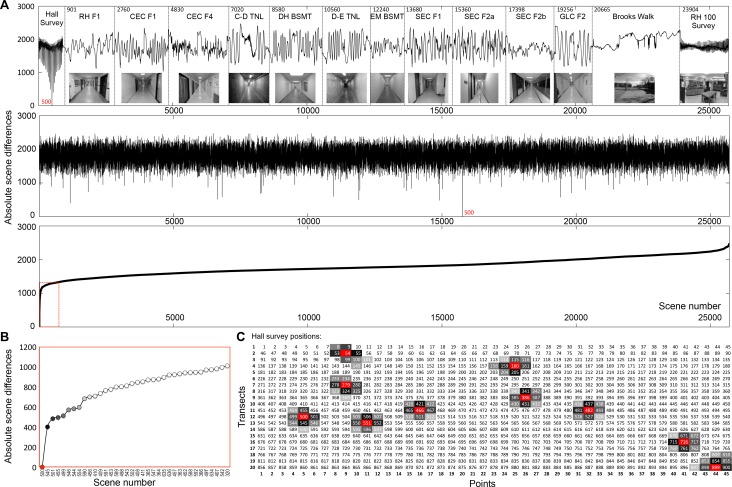
Comparison of hall survey images to all corridor and outdoor scenes. **A** All scenes were extracted from the corridor and outdoor videos (30 frames per second, converted to 100 levels of gray, enhanced via histeq, circularized, and pixelated to 100x100). The scenes were stacked in a MATLAB structured matrix and bracketed at the beginning with the 900 hall and 1800 laboratory survey scenes. Each corridor and external video (other than RH F1) consisted of two videos traversing the length in each direction. In all, the matrix consisted of 25,703 scenes. The top trace shows SAD comparisons of all scenes to scene 500 (which is within the hall survey set). The middle trace is a randomized plot of the same result (scene 500 is indicated in red). The lower trace is a rank-ordered plot of the same result. The values within the dashed red box are expanded in **B** and relevant scene numbers from the unsorted matrix are indicated on the X-axis. The markers are color coded, with darker gray levels indicating greater image similarity. These shades are carried over to **C,** where gray-shaded boxes represent the 8 most similar scenes around scene 500 (left-most red box). The other sets of boxes in C represent 9 additional, randomly selected focal scenes (red) taken from the 900 hall survey scenes and subjected to the same analysis; their 8 most similar neighbors are indicated in gray.

Finally, we tested whether there was any aliasing between all corridor, laboratory, and outdoor scenes compared to our 900-scene, two-dimensional corridor survey. We compiled one large matrix of >25,000 images comprising all scenes taken from the corridor videos (2 sets of scenes from each corridor, one set obtained for each from both directions, except for RH F1), laboratory and outdoor settings. Scenes were organized linearly and grouped by setting (Fig 12A, top section). We chose 10 random scenes from our 2D corridor survey and calculated the SAD values of these scenes compared to all other scenes in the matrix (Fig 12) to assess whether aliasing between these points was possible; (Fig 12A and 12B) shows the analysis for point 500. No scene from any other corridor or setting, including the adjacent corridor and laboratory survey was similar to scene 500 in the matrix. Furthermore, the scenes closest in visual information (lowest absolute scene difference value) were those scenes immediately adjacent to it (Hall Survey section in top trace, Fig 12B and 12C). The scenes from the rest of the corridors in the matrix were not within 1000 absolute scene difference values (Fig 12A). The results of a similar analysis for the additional nine focal scenes are shown in Fig 12C. As shown for scene 500, the eight scenes with the lowest absolute scene difference value to each of the additional nine focal scenes were also adjacent scenes (Fig 12C in gray shades).

## Discussion

There are many solutions for navigation, including fusion of numerous sensors, such as LIDAR, GPS and vision, as well as simultaneous localization and mapping (SLAM) algorithms. While effective, many of these solutions are computationally intensive and/or expensive. We believe there is value in examining nature for insights into mechanisms for navigation. Natural selection has sculpted the navigational capabilities of insects into simple, efficient, and highly effective navigational toolsets. In many ways, bees and ants still outperform the today’s top-of-the-line UAV technologies. They can navigate multiple kilometers from hive to food source and back with minimal brain capacity and without external GPS assistance. They also have other capabilities such as path integration and polarized light E-vector recognition for estimating direction and distance. While it may be possible to add odometry and SLAM technologies, perhaps as a means to establish path integration, our current interests were to explore the navigational limits of using previously experienced visual information for guidance along complex paths by way of the insect inspired scene familiarity algorithm.

The *Navigation by Scene Familiarity Hypothesis* suggests that panoramic scenes experienced by navigating insects as they move on a training path are processed by dense matrices of sensors and stored as unique synaptic engrams within networks of neurons between the retinae and the central complex [14, 16, 18]. Since each unique visual pattern projected on the insect’s compound eyes addresses a particular memory, the animal can retrace the original path by simply scanning the environment and moving towards the scene that appears most familiar at any moment. The scenes do not need to be stored in any particular order, since traversing the previously experienced environment calls up the appropriate memory.

An important assumption of this hypothesis is that scenes in the animal’s visual environment are distinct enough to guide navigation without aliasing. Both natural scenes and indoor environments have been shown to hold sufficient information for navigation using reference points and SLAM based algorithms [10, 21, 23–26]. We were interested in testing whether an indoor environment contained enough visual information for a scene familiarity algorithm to recapitulate training paths, without using any SLAM or reference points. We made a high-density visual survey of our laboratory at one plane by using a rail system that spanned the lab at a fixed height, 1.28 m, above the floor. From this vantage point, many items provided visual information, including cabinets, bookshelves, windows, lights, and a chalkboard. Our laboratory’s visual landscape consisted of 1800 panoramic snapshots in a regular grid that we pixelated and circularized for comparisons with other scenes at various rotations. We used this pre-sampled visual environment to systematically alter sensor parameters such as resolution and degree of rotation for robust analysis of the effects on route recapitulation. The landscape information analysis of Fig 3 shows significant differences among the scenes in the room. With a 100x100 sensor (10 gray levels), even the lowest mean differences (middle of the room) are substantial, suggesting that abundant visual information exists for navigation throughout the room. Volcano plots (such as Fig 5A) that compare single scenes to all other room scenes show steep average image differences, again highlighting the significant amount of visual information. Also, the image differences for routes consisting of a series of linked images form distinct troughs of familiarity when compared to all other room scenes (Fig 5B, 5C and 5D). These plots indicate that the scenes with the minimum image difference value compared to a reference scene are those immediately adjacent to it; no other scene shares enough similarity with the focal scene to misdirect the agent. Combined, these results indicate that our laboratory has sufficient information to provide distinct views for an agent to recapitulate a previously experienced route.

Up to now, most work on visual navigation algorithms based on scene familiarity have used a fixed sensor size and rotational degree or have not directly tested differences in these variables on path recapitulation [14–19]. Previous work has also suggested that very low sensor resolutions can allow for successful place recognition and navigation in certain situations [18, 21]. Optimizing the sensor resolution and/or degree of rotation could improve performance or reduce the computation time of path recapitulation. We analyzed the quality of visual information by examining the translational (IDF) and rotational (RIDF) information available for sensors of various densities. In general, the width of translational and rotational catchment areas decreases with increased sensor resolution. For example, the width of the translational catchment area for a 10x10 sensor midway up the catchment slope (p50) is about 1.8 m (= 2x0.9 m; see Fig 4B), whereas the width falls to less than a meter with resolutions denser than 30x30. Similarly, lower sensor densities accommodate wider angles of sensor rotation compared to higher density sensors. For example, the span of rotation at the p50 familiarity level is about 26 degrees (= 2x13°) for the 10x10 sensor and constricts to less than 15 degrees for sensor densities greater than 30x30.

Given these sensor characteristics, we would expect the performance of our auto-tracking algorithm to be contingent upon both the pixel density and the rotation interval. This prediction is confirmed by the route recapitulations of Fig 7, where agents with lower-density sensors recovered the training path from farther displacement distances compared to higher-density sensors, but tracked the training path with less precision once within the catchment valley.

Complex routes likely pose significantly more difficult situations for the auto-tracking algorithm, such as larger-degree turns, more views overlapping with learned path images in the background, and greater distances to be covered. Because of these characteristics, the tracking performance for a complex route should not be as consistent as it is for a simple route. In Fig 8, we used a complex training path to assess tracking ability while varying pixel density, rotation interval, and familiarity threshold. We found trade-offs in tracking accuracy and speed; we also found that specific features of the training path affected performance. For example, the constriction of the training path at the top of the “O” induced aliasing for the 10x10, 20x20, and 40x40 sensors at 1° rotation interval and familiarity threshold of 2, but not for the 80x80 sensor. Also, the 40x40 sensor had only four successful recapitulations, all at the 2° rotation interval, while the 80x80 sensor had six successful recapitulations scattered across 1-3° rotation intervals and familiarity thresholds from 3 to 6. In general, the 80x80 sensor tracked very closely to the training path, but required more inspection points and therefore took longer to complete its recapitulations. The fastest recapitulation (T = 160) was for the 40x40 sensor at 2° rotation interval and a familiarity threshold of 2, but the path departure was large (D = 23.2m). The recapitulation with the largest departure (D = 32.4m) was for the 20x20 sensor, also at 2° rotation interval and a familiarity threshold of 2 (its recapitulation time was T = 178). It is intriguing to consider how sets of sensor parameters could be analogous to natural selection of insect sensor attributes in certain environments and visual landscapes. It may also be possible to develop a learning module for specific environments and use those parameters to establish navigation criteria for an agent within that environment.

One of the major tasks of route recapitulation in nature is navigating to an endpoint that was not in view at the start of the path. It has been proposed that bees and ants use several mechanisms to achieve this feat, such as landmark recognition and sequentially completing routes between landmarks. We tested a complex path with our route recapitulation algorithm. Upon extending our visual landscape to include images of our doorway and adjacent corridor, we generated a complex meandering path beginning in the laboratory and finishing in the adjacent corridor with a wall blocking the view of the endpoint. This path was easily recapitulated using our algorithm. Furthermore, the route taken by the agent only deviated from the training path at some of the sharp turns.

We were also interested in whether a novel training path generated by moving a cart with a camera attached throughout our laboratory could be recapitulated using our algorithm and scene landscape. It is worth remembering that our visual landscape and training routes were conducted in a systematic way using train tracks for reference and our training routes were based on linked surveyed images. When tested at varying sensor resolutions, a novel U-shaped training path (not linked surveyed images) was successfully recapitulated with surprising accuracy, even though the training path was generated approximately eight months after the image landscape was created. The accuracy of the recapitulations was influenced by the sensor resolution. A more accurate recapitulation of the learned path was achieved with higher sensor resolutions as compared to the 10x10 sensor. The sparse nature of our image landscape (surveyed images every 12.7 cm) may have influenced the accuracy of recapitulation. A denser survey should produce a more accurate recapitulation, even at 10x10 and 5x5 resolution.

Outdoor scenes, especially those in urban settings and with numerous objects along the horizon, possess abundant visual information. The likelihood of scene aliasing is very low in these settings (see Fig 11, Brooks Walk, for an example). However, indoor homogeneous environments with repetitive features such as equally spaced doors or featureless walls offer much higher potential for aliasing. This is not a challenge for insects such as bees or ants, which are moving toward a goal such as a food source or nest/hive in non-homogenous outdoor environments with abundant visual information (as in Fig 11, Brooks Walk). They can also access additional information from path integration and learning flights/walks around the goal site from all directions. Nevertheless, we were interested in whether any scene that we captured in our lab or adjacent corridor was similar to any other corridor or outdoor scene. Most of the corridors had local minima present (Fig 11). This is not surprising given the repetitive nature of these environments. No sum of absolute differences (SAD) between scenes fell to zero for any of the corridors suggesting that no two scenes were identical. Of course, an SAD of zero is unlikely to occur, even if the sensor were placed at the same position, especially with a 100 x 100 pixel resolution.

It is possible that scenes from featureless or identically structured laboratories or corridors could be deemed identical with a vision-centric scene familiarity algorithm. We suggest that many other navigational strategies would also fail to recapitulate training paths in this setting. Aliasing could occur from a few of the tested corridors in our study using our scene familiarity algorithm, especially those with the lowest rank in Fig 11, such as GLC-F2 and CEC F4. For instance, GLC-F2 is a corridor with white walls, few objects on the walls, and multiple office doors. If an agent were trained along a path from the beginning of the GLC-F2 corridor to the first door and then placed farther down the hallway (beyond the training path door entrance) for recapitulation, it could move forward along the center of the corridor and into the next door instead of turning around and returning to the correct door. Incorporating other features into our algorithm should improve tracking accuracy in these rare situations. For example, we could loop a portion of the output of a previous scene recognition event (scene 1) with the input of the subsequent scene recognition event (scene 2); in this scenario, scene 2 would only be only be recognized in the context of scene 1. This addition could improve accuracy, as the decision to move forward is weighted by previous images. Another strategy that has improved accuracy in recapitulation with scene familiarity is incorporating multiple short paths that collect images of the goal location from all directions, analogous to learning walks, into the training path acquisition [14].

Our results indicate that a systematic visual scan of an indoor environment contains substantially distinct translational and rotational visual information. This information is sufficient to allow route recapitulation, even when the visual sensor has a low resolution (10x10 pixels) and the path endpoint is not visible from the beginning. Given that our algorithm allows for both translational and rotational comparisons with previously learned images, it easily finds the best direction to travel at each point. Furthermore, the side-to-side scanning or saccading allows the agent to easily slide into the major visual information catchment areas of the training path. Algorithms based on navigation by scene familiarity thus provide a simple solution to route learning and recapitulation, especially over long distances. There are major benefits to this approach. For instance, it can be used in the absence of GPS and does not rely on odometric data, sequential ordering of stored images, or storage of waypoints. It is important to remember that our visual scan of the environment was conducted inside a lighted laboratory. In nature, the movement of the sun across the sky creates shadows that change throughout the day; this may affect the performance of the algorithm. However, pre-processing methods have been applied to images in IDF navigation algorithms that allow recapitulation even in varying light levels [19]. A visual scan of the environment during multiple times of day could also provide enough stored images, which could be used for recapitulation of routes. Interestingly, evidence suggests that bees possess a spatiotemporal memory, which facilitates navigation to foraging sites during specific times of the day [27, 28]. Coding learned routes with certain times of day or the use of a sun sensor could facilitate navigation during different times of the day in outdoor settings.

This study was conducted on one vertical plane, at approximately 1.28 meters above the floor. The success of route recapitulation at different heights within our landscape is important for use in flying UAVs. We believe that video captured at multiple heights and orientations with a panoramic or omnidirectional camera will provide enough visual information for successful route recapitulation by UAVs. Interestingly, vision and IDF approaches have recently been implemented in local homing and vertical takeoff and landing in UAVs [11]. We would like to extend this work to use the scene familiarity approach to recapitulate complex visual pathways in three-dimensional space.

## Supporting Information

S1 Video GUI and video of route recapitulations from Fig 8 with a 10x10 sensor, 1 degree rotational interval and a threshold of 3. Video of an unsuccessful route recapitulation with a moderate distance covered. With these settings, the agent was unable to make the sharp corner at the end of the “O” and thus returned to the beginning and began retracing the “O” path.
(MP4)

S2 Video GUI and video of route recapitulations from Fig 8 with a 10x10 sensor, 1 degree rotational interval and a threshold of 4. Video of a successful route recapitulation with a moderate distance covered. Interestingly, the only difference in this video from S1 is the threshold value of 4 instead of 3. With this change, the agent was able to round the sharp corner and successfully finish the path instead of continuing around the “O”.
(MP4)

S3 Video GUI and video of route recapitulations from Fig 8 with a 20x20 sensor, 2 degree rotational interval and a threshold of 2. Video of a successful route with a long distance covered.
(MP4)

S4 Video GUI and video of route recapitulations from Fig 8 with an 80x80 sensor, 1 degree rotational interval and a threshold of 3. Video of an unsuccessful route recapitulation that failed to make the sharp turn in the “U” letter.
(MP4)

S5 Video GUI and video of route recapitulations from Fig 8 with an 80x80 sensor, 1 degree rotational interval and a threshold of 4. Video of a successful route recapitulation, which only differed from the S1 Video 4 by an increased threshold value of 1. Note the distance covered was very small but the time required for recapitulation was long.
(MP4)

S6 Video GUI and video of a complex meandering path from the laboratory to the corridor from Fig 9. Video highlighting a successful route recapitulation of a complex meandering path from the inside of the laboratory and finishing at a point obstructed from the starting view in the adjacent corridor.
(M4V)

S7 Video Depth of the catchment area and the image difference values present in the complex path recapitulation from the lab to the corridor in Fig 9. A movie highlighting a close up view of the catchment area and the image differences present in the successful recapitulation of the training path from the laboratory to the corridor.
(MOV)

S8 Video GUI and video of a novel training path from Fig 10 with a 5x5 sensor. Video highlighting an unsuccessful route recapitulation of a novel training path (not a sequential set of landscape images) at a resolution of 5x5 and 1 degree of rotational interval. The recapitulation begins well and remains on track during the first straightaway but stops during the first turn encountered.
(MP4)

S9 Video GUI and video of a novel training path from Fig 10 with a 10x10 sensor. Video highlighting a successful route recapitulation of a novel training path (not a sequential set of landscape images) at a resolution of 10x10 and 1 degree of rotational interval. Note the completion of the route recapitulation despite the difficulty rounding the corners in the “U”.
(MP4)

S10 Video GUI and video of a novel training path from Fig 10 with a 20x20 sensor. Video highlighting a successful route recapitulation of a novel training path (not a sequential set of landscape images) at a resolution of 20x20 and 1 degree of rotational interval. Note the noticeable easier time rounding the corners in the “U” path.
(MP4)

S11 Video GUI and video of a novel training path from Fig 10 with a 40x40 sensor. Video highlighting a successful route recapitulation of a novel training path (not a sequential set of landscape images) at a resolution of 40x40 and 1 degree of rotational interval.
(MP4)

S12 Video GUI and video of a novel training path from Fig 10 with a 100x100 sensor. Video highlighting a successful route recapitulation of a novel training path (not a sequential set of landscape images) at a resolution of 100x100 and 1 degree of rotational interval.
(MP4)
